# *catena*-Poly[[*trans*-bis­(1,3-benzo­thia­zole-κ*N*)manganese(II)]-di-μ-chlorido]

**DOI:** 10.1107/S1600536814014159

**Published:** 2014-06-21

**Authors:** Hasna Bouchareb, Sabrina Benmebarek, Sofiane Bouacida, Hocine Merazig, Mhamed Boudraa

**Affiliations:** a Unité de Recherche de Chimie de l’Environnement et Moléculaire Structurale, CHEMS, Université Constantine 1, 25000, Algeria; b Département Sciences de la Matière, Faculté des Sciences Exactes et Sciences de la Nature et de la Vie, Université Oum El Bouaghi, Algeria

**Keywords:** crystal structure

## Abstract

In the title coordination polymer, [MnCl_2_(C_7_H_5_NS)_2_]_*n*_, the Mn^II^ ion is located on the inter­section of a twofold rotation axis and a mirror plane and adopts an octa­hedral coordination geometry defined by two mutually *trans* N atoms from benzo­thia­zole ligands which occupy the axial positions, and four Cl atoms which form the equatorial sites. The Mn^II^ ions are connected by two bridging Cl atoms, forming chains parallel to the *c* axis. The crystal packing can be descibed as alternating layers parallel to (001) featuring π–π stacking inter­actions with a centroid–centroid distance of 3.6029 (15) Å.

## Related literature   

For applications of benzo­thia­zole and its derivatives, see: Petkova *et al.* (2000 ▶); Karisson *et al.* (2003 ▶); Khan *et al.* (2011 ▶). For related structures see: Bouchareb *et al.* (2013 ▶); Roh & Jeong (2007 ▶); Popović *et al.* (2003 ▶); Maniukiewicz (2004 ▶).
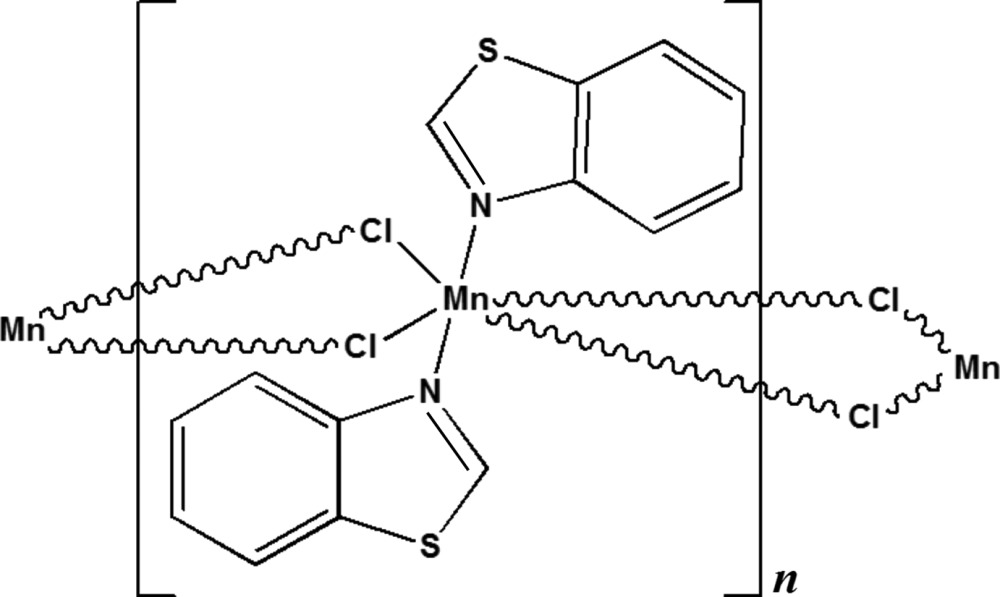


## Experimental   

### 

#### Crystal data  

[MnCl_2_(C_7_H_5_NS)_2_]
*M* *_r_* = 396.22
Tetragonal, 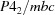

*a* = 14.761 (6) Å
*c* = 7.170 (3) Å
*V* = 1562.3 (14) Å^3^
*Z* = 4
Mo *K*α radiation
μ = 1.45 mm^−1^
*T* = 150 K
0.19 × 0.14 × 0.12 mm

#### Data collection  

Bruker APEXII diffractometer
Absorption correction: multi-scan (*SADABS*; Sheldrick, 2002 ▶) *T*_min_ = 0.674, *T*_max_ = 0.746
15714 measured reflections
918 independent reflections
685 reflections with *I* > 2σ(*I*)
*R*_int_ = 0.117

#### Refinement  

*R*[*F* ^2^ > 2σ(*F* ^2^)] = 0.042
*wR*(*F* ^2^) = 0.092
*S* = 1.14
918 reflections
64 parameters
H-atom parameters constrained
Δρ_max_ = 0.55 e Å^−3^
Δρ_min_ = −0.46 e Å^−3^

### 

Data collection: *APEX2* (Bruker, 2011 ▶); cell refinement: *SAINT* (Bruker, 2011 ▶); data reduction: *SAINT*; program(s) used to solve structure: *SIR2002* (Burla *et al.*, 2005 ▶); program(s) used to refine structure: *SHELXL97* (Sheldrick, 2008 ▶); molecular graphics: *ORTEP-3 for Windows* (Farrugia, 2012 ▶) and *DIAMOND* (Brandenburg & Berndt, 2001 ▶); software used to prepare material for publication: *WinGX* (Farrugia, 2012 ▶).

## Supplementary Material

Crystal structure: contains datablock(s) I. DOI: 10.1107/S1600536814014159/lh5716sup1.cif

Structure factors: contains datablock(s) I. DOI: 10.1107/S1600536814014159/lh5716Isup2.hkl

CCDC reference: 1008670

Additional supporting information:  crystallographic information; 3D view; checkCIF report

## Figures and Tables

**Fig. 1.**
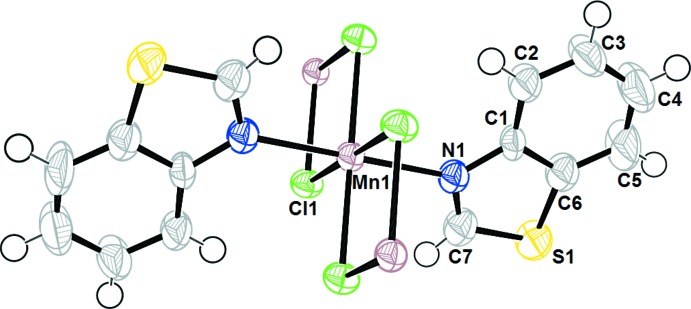
The molecular structure of, (I), with displacement ellipsoids drawn at the 50% probability level. Only the contents of the asymmetric unit are numbered. H atoms are represented as small spheres of arbitrary radii.

**Fig. 2.**
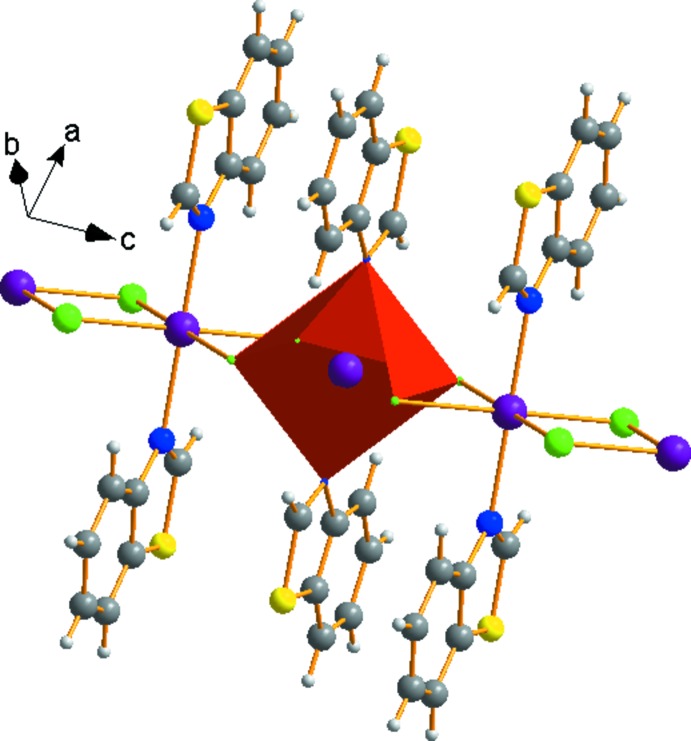
The coordination around the MnII ion in a chain of octahedra parallel to the c axis

**Fig. 3.**
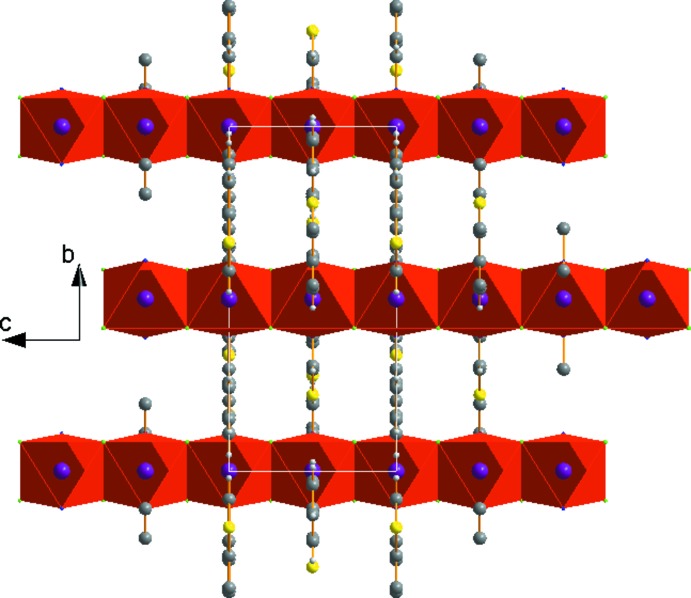
Packing diagram of (I) viewed along the a axis showing alterning layers parallel to (001).

## supplementary crystallographic information

### S1. Comment

In recent years, benzothiazole and its derivatives have attracted more attention because they exhibit interesting optical and biological activities (Petkova *et al.*, 2000; Karisson *et al.*, 2003; Khan *et al.* 2011). Related structural studies are partly focused on the fact that the benzothiazole ring contains N, S and O as potential donor atoms, which exhibit good coordination capacity, and so are propitious to build novel complexes (Roh *et al.* 2007, Popović *et al.* 2003, Maniukiewicz 2004). As part of our ongoing studies of benzothiazole-based coordination networks (Bouchareb *et al.* 2013), we report herein the structure of a coordination polymer of manganese and a benzothiazole ligand (I). The molecular geometry and the atom-numbering scheme are shown in Fig 1.

In the title compound, the Mn^II^ cation is located on the intersection of a twofold rotation axis and a mirror plane. The coordination sphere is defined by two mutually *trans* N atoms from two neutral monodentate benzothiazole ligands occupying the axial positions, and four Cl atoms lying in the equatorial plane. All Mn–Cl bond lengths are identical [2.5232 (10)] Å by symmetry and the Mn—N bond lengths are 2.307 (4) Å. In the coordination octahedron, all N—Mn—Cl and Cl—Mn—Cl bond angles are in the range of 89.40 (7) – 90.60 (7)°. The Mn^II^ ions are connected by two bridging Cl atoms, resulting in a chain of octahedra parallel to the *c* axis (Fig. 2). The crystal packing can be descibed as alterning layers parallel to (001) (Fig. 3). The crystal structure features two π–π stacking interactions: *Cg*1—*Cg*1 = 3.6029 (15) Å and *Cg*1—*Cg*2 = 4.048 (2) Å, Where, *Cg*1 is the centroid of the imidazole ring (N1/C7/S1/C6/C1) and *Cg*2 is the centroid of the fused benzene ring (C1/C2/C3/C4/C5/C6). No hydrogen bonds are observed in the structure.

### S2. Experimental

Benzothiazole (1 ml) and ethanol (1 ml) were added to a solution of MnCl_2_·4H_2_O (39.5 mg, 0.2 mmol) in water (10 ml). The mixture was then refluxed with stirring for 3 h and the resulting solution was left to stand at room temperature. After several days, single crystals suitable for X-ray diffraction were obtained.

### S3. Refinement

All non-H atoms were refined with anisotropic displacement parameters. Approximate positions for all H atoms were first obtained from the difference electron density map. However, the H atoms were placed in idealized positions and refined in a riding-model approximation. The applied constraints were as follow: C—H = 0.93 Å and *U*_iso_ = 1.2*U*_eq_(C).

### Crystal data

**Table:**

[MnCl_2_(C_7_H_5_NS)_2_]	*D*_x_ = 1.685 Mg m^−^^3^
*M**_r_* = 396.22	Mo *K*α radiation, λ = 0.71073 Å
Tetragonal, *P*4_2_/*m**b**c*	Cell parameters from 1270 reflections
Hall symbol: -P 4c 2ab	θ = 3.1–25.2°
*a* = 14.761 (6) Å	µ = 1.45 mm^−^^1^
*c* = 7.170 (3) Å	*T* = 150 K
*V* = 1562.3 (14) Å^3^	Block, colorless
*Z* = 4	0.19 × 0.14 × 0.12 mm
*F*(000) = 796	

### Data collection

**Table:**

Bruker APEXII diffractometer	918 independent reflections
Radiation source: sealed tube	685 reflections with *I* > 2σ(*I*)
Graphite monochromator	*R*_int_ = 0.117
φ and ω scans	θ_max_ = 27.0°, θ_min_ = 2.8°
Absorption correction: multi-scan (*SADABS*; Sheldrick, 2002)	*h* = −18→18
*T*_min_ = 0.674, *T*_max_ = 0.746	*k* = −18→18
15714 measured reflections	*l* = −9→9

### Refinement

**Table:**

Refinement on *F*^2^	Primary atom site location: structure-invariant direct methods
Least-squares matrix: full	Secondary atom site location: difference Fourier map
*R*[*F*^2^ > 2σ(*F*^2^)] = 0.042	Hydrogen site location: inferred from neighbouring sites
*wR*(*F*^2^) = 0.092	H-atom parameters constrained
*S* = 1.14	*w* = 1/[σ^2^(*F*_o_^2^) + (0.0376*P*)^2^ + 1.2805*P*] where *P* = (*F*_o_^2^ + 2*F*_c_^2^)/3
918 reflections	(Δ/σ)_max_ = 0.007
64 parameters	Δρ_max_ = 0.55 e Å^−^^3^
0 restraints	Δρ_min_ = −0.46 e Å^−^^3^

### Special details

**Table:**

Geometry. All e.s.d.'s (except the e.s.d. in the dihedral angle between two l.s. planes) are estimated using the full covariance matrix. The cell e.s.d.'s are taken into account individually in the estimation of e.s.d.'s in distances, angles and torsion angles; correlations between e.s.d.'s in cell parameters are only used when they are defined by crystal symmetry. An approximate (isotropic) treatment of cell e.s.d.'s is used for estimating e.s.d.'s involving l.s. planes.
Refinement. Refinement of *F*^2^ against ALL reflections. The weighted *R*-factor *wR* and goodness of fit *S* are based on *F*^2^, conventional *R*-factors *R* are based on *F*, with *F* set to zero for negative *F*^2^. The threshold expression of *F*^2^ > σ(*F*^2^) is used only for calculating *R*-factors(gt) *etc*. and is not relevant to the choice of reflections for refinement. *R*-factors based on *F*^2^ are statistically about twice as large as those based on *F*, and *R*- factors based on ALL data will be even larger.

### Fractional atomic coordinates and isotropic or equivalent isotropic displacement parameters (Å^2^)

**Table:**

	*x*	*y*	*z*	*U*_iso_*/*U*_eq_	
C1	0.8908 (3)	0.2962 (3)	1	0.0160 (9)	
C2	0.9693 (3)	0.2427 (3)	1	0.0242 (11)	
H2	1.0264	0.2692	1	0.029*	
C3	0.9597 (3)	0.1501 (3)	1	0.0337 (13)	
H3	1.0112	0.1137	1	0.04*	
C4	0.8742 (3)	0.1096 (3)	1	0.0423 (16)	
H4	0.8697	0.0468	1	0.051*	
C5	0.7963 (3)	0.1612 (3)	1	0.0398 (15)	
H5	0.7395	0.134	1	0.048*	
C6	0.8050 (3)	0.2547 (3)	1	0.0226 (10)	
C7	0.8052 (3)	0.4183 (3)	1	0.0225 (11)	
H7	0.7909	0.4796	1	0.027*	
N1	0.8879 (2)	0.3912 (2)	1	0.0167 (7)	
S1	0.72177 (8)	0.33651 (9)	1	0.0284 (3)	
Cl1	0.91494 (5)	0.58506 (5)	0.75	0.0163 (2)	
Mn1	1	0.5	1	0.0135 (2)	

### Atomic displacement parameters (Å^2^)

**Table:**

	*U*^11^	*U*^22^	*U*^33^	*U*^12^	*U*^13^	*U*^23^
C1	0.018 (2)	0.012 (2)	0.018 (2)	−0.0047 (18)	0	0
C2	0.014 (2)	0.018 (2)	0.041 (3)	−0.0029 (18)	0	0
C3	0.018 (2)	0.016 (2)	0.067 (4)	0.002 (2)	0	0
C4	0.026 (3)	0.016 (3)	0.084 (5)	−0.004 (2)	0	0
C5	0.020 (3)	0.023 (3)	0.077 (4)	−0.012 (2)	0	0
C6	0.018 (2)	0.020 (2)	0.030 (3)	−0.0039 (19)	0	0
C7	0.019 (2)	0.023 (3)	0.026 (3)	−0.004 (2)	0	0
N1	0.015 (2)	0.016 (2)	0.0192 (18)	−0.0017 (14)	0	0
S1	0.0122 (6)	0.0245 (7)	0.0484 (8)	−0.0023 (5)	0	0
Cl1	0.0161 (3)	0.0161 (3)	0.0167 (5)	0.0030 (4)	0.0004 (4)	0.0004 (4)
Mn1	0.0128 (5)	0.0128 (5)	0.0150 (4)	−0.0003 (3)	0	0

### Geometric parameters (Å, º)

**Table:**

C1—C2	1.402 (6)	C6—S1	1.723 (5)
C1—N1	1.402 (5)	C7—N1	1.284 (6)
C1—C6	1.406 (6)	C7—S1	1.724 (5)
C2—C3	1.374 (6)	C7—H7	0.93
C2—H2	0.93	N1—Mn1	2.307 (4)
C3—C4	1.397 (7)	Cl1—Mn1^i^	2.5232 (10)
C3—H3	0.93	Cl1—Mn1	2.5232 (10)
C4—C5	1.379 (7)	Mn1—N1^ii^	2.307 (4)
C4—H4	0.93	Mn1—Cl1^iii^	2.5232 (10)
C5—C6	1.386 (7)	Mn1—Cl1^ii^	2.5232 (10)
C5—H5	0.93	Mn1—Cl1^iv^	2.5232 (10)
			
C2—C1—N1	126.1 (4)	C7—N1—C1	109.9 (4)
C2—C1—C6	119.9 (4)	C7—N1—Mn1	117.7 (3)
N1—C1—C6	114.1 (4)	C1—N1—Mn1	132.4 (3)
C3—C2—C1	118.4 (4)	C6—S1—C7	88.9 (2)
C3—C2—H2	120.8	Mn1^i^—Cl1—Mn1	90.54 (4)
C1—C2—H2	120.8	N1^ii^—Mn1—N1	180
C2—C3—C4	121.2 (5)	N1^ii^—Mn1—Cl1^iii^	89.40 (7)
C2—C3—H3	119.4	N1—Mn1—Cl1^iii^	90.60 (7)
C4—C3—H3	119.4	N1^ii^—Mn1—Cl1^ii^	89.40 (7)
C5—C4—C3	121.1 (5)	N1—Mn1—Cl1^ii^	90.60 (7)
C5—C4—H4	119.4	Cl1^iii^—Mn1—Cl1^ii^	90.54 (4)
C3—C4—H4	119.4	N1^ii^—Mn1—Cl1	90.60 (7)
C4—C5—C6	118.2 (5)	N1—Mn1—Cl1	89.40 (7)
C4—C5—H5	120.9	Cl1^iii^—Mn1—Cl1	89.46 (4)
C6—C5—H5	120.9	Cl1^ii^—Mn1—Cl1	180
C5—C6—C1	121.1 (4)	N1^ii^—Mn1—Cl1^iv^	90.60 (7)
C5—C6—S1	129.2 (4)	N1—Mn1—Cl1^iv^	89.40 (7)
C1—C6—S1	109.7 (3)	Cl1^iii^—Mn1—Cl1^iv^	180
N1—C7—S1	117.4 (4)	Cl1^ii^—Mn1—Cl1^iv^	89.46 (4)
N1—C7—H7	121.3	Cl1—Mn1—Cl1^iv^	90.54 (4)
S1—C7—H7	121.3		
			
N1—C1—C2—C3	180	C6—C1—N1—Mn1	180
C6—C1—C2—C3	0	C5—C6—S1—C7	180
C1—C2—C3—C4	0	C1—C6—S1—C7	0
C2—C3—C4—C5	0	N1—C7—S1—C6	0
C3—C4—C5—C6	0	C7—N1—Mn1—Cl1^iii^	−134.72 (2)
C4—C5—C6—C1	0	C1—N1—Mn1—Cl1^iii^	45.28 (2)
C4—C5—C6—S1	180	C7—N1—Mn1—Cl1^ii^	134.72 (2)
C2—C1—C6—C5	0	C1—N1—Mn1—Cl1^ii^	−45.28 (2)
N1—C1—C6—C5	180	C7—N1—Mn1—Cl1	−45.28 (2)
C2—C1—C6—S1	180	C1—N1—Mn1—Cl1	134.72 (2)
N1—C1—C6—S1	0	C7—N1—Mn1—Cl1^iv^	45.28 (2)
S1—C7—N1—C1	0	C1—N1—Mn1—Cl1^iv^	−134.72 (2)
S1—C7—N1—Mn1	180	Mn1^i^—Cl1—Mn1—N1^ii^	89.39 (7)
C2—C1—N1—C7	180	Mn1^i^—Cl1—Mn1—N1	−90.61 (7)
C6—C1—N1—C7	0	Mn1^i^—Cl1—Mn1—Cl1^iv^	180
C2—C1—N1—Mn1	0		

Symmetry codes: (i) *y*+1/2, *x*−1/2, −*z*+3/2; (ii) −*x*+2, −*y*+1, −*z*+2; (iii) −*x*+2, −*y*+1, *z*; (iv) *x*, *y*, −*z*+2.
